# *Giardia duodenalis* Colonization Slightly Affects Gut Microbiota and Hematological Parameters in Clinically Healthy Dogs

**DOI:** 10.3390/ani13060958

**Published:** 2023-03-07

**Authors:** Arianna Peruzzo, Marta Vascellari, Andrea Massaro, Marzia Mancin, Annalisa Stefani, Massimiliano Orsini, Patrizia Danesi, Sara Petrin, Antonio Carminato, Michele Matteo Santoro, Roberto Speranza, Carmen Losasso, Gioia Capelli

**Affiliations:** 1 Laboratory of Microbial Ecology and Genomics, Istituto Zooprofilattico Sperimentale delle Venezie, 35020 Legnaro, Italy; 2 Laboratory of Histopathology, Istituto Zooprofilattico Sperimentale delle Venezie, 35020 Legnaro, Italy; 3 Laboratory of Enhancement of Food Production, Istituto Zooprofilattico Sperimentale delle Venezie, 35020 Legnaro, Italy; 4 Laboratory of Animal Medicine, Istituto Zooprofilattico Sperimentale delle Venezie, 35020 Legnaro, Italy; 5 Laboratory of Parasitology, Mycology and Medical Entomology, Istituto Zooprofilattico Sperimentale delle Venezie, 35020 Legnaro, Italy; 6 Corso Allevamento e Addestramento Cinofili, Guardia di Finanza, 06061 Castiglione del Lago, Italy

**Keywords:** dogs, *Giardia duodenalis*, gut, lipid metabolism, microbiota

## Abstract

**Simple Summary:**

*Giardia* is a worldwide cause of acute diarrheal disease both in humans and animals. Domestic dogs may be either asymptomatic or may show clinical signs. Here, we report a study comparing two groups of clinically healthy German shepherd dogs differing for *G. duodenalis* colonization. Gut microbiota and the hematological, biochemical, and fecal parameters related to intestinal function were investigated. The results display a scenario in which *G. duodenalis* exerts an effect upon the gut microbiota affecting the proportion of a few bacterial taxa known to be associated with improved lipids metabolism and protection from gut inflammation. This also suggests that the antiparasitic treatments that are usually administered to *G. duodenalis*-positive dogs might be avoided in clinically healthy subjects since the presence of *G. duodenalis* does not substantially modify the microbial ecology of the intestinal lumen nor the hematological markers of disease.

**Abstract:**

*Giardia duodenalis* (*Giardia*) is a worldwide cause of acute diarrheal disease both in humans and animals. The primary aim of this study was to investigate possible variations in gut microbiota in a population of asymptomatic dogs (*n* = 31), naturally infected or not by *Giardia*. Gut microbiota and the hematological, biochemical, and fecal parameters related to intestinal function were investigated. *Giardia* infection was associated with a significant shift of beta diversity, showing a relevant reduction of Gammaproteobacteria and an increase of Fusobacteria in male-positive dogs if compared with negatives. A significant imbalance of different bacterial taxa, with particular reference to the *Erysipelotrichales*, *Lactobacillales*, *Clostridiales*, and *Burkholderiales* orders, was observed, with the first two being higher in *Giardia*-positive dogs. *Giardia*-positive males displayed significantly higher values of cCRP than negative males as well as positive females, supporting the presence of a pro-inflammatory state. Taken together, these results indicate that the presence of *Giardia* does not substantially modify the microbial ecology of the intestine nor the hematological markers of disease. Thus treatments against *Giardia* should be considered with caution in asymptomatic subjects.

## 1. Introduction

*Giardia duodenalis* (*Giardia*) is a common worldwide parasite of both humans and domestic animals, and it is currently recognized as the most prevalent gastrointestinal parasite in domestic dogs, closely followed by hookworms and coccidian [1,2,3,4,5]. The role of *Giardia* in causing a broad range of clinical manifestations, from asymptomatic to acute/chronic diarrheal disease, remains a matter of debate. Even though *Giardia* is frequently detected in diarrheic animals, particularly in puppies, many hosts remain asymptomatic despite shedding high numbers of environmentally resistant cysts [6]. The main sustained hypothesis on giardiosis and associated sequelae is that parasite attachment causes the loss of epithelial barrier function [7,8], favoring the penetration of intestinal bacteria into the inflamed intestinal wall, resulting in permanent damage to the intestinal epithelium [9]. Some authors postulated that changes in the resident intestinal microflora are responsible for the disease outcome of giardiosis [10]. However, the host–parasite interaction is not a one-way process, and changes in the host microbiome itself may favor the contact between parasites and host cells. Such changes may be caused by different stresses, such as nutritional or environmental changes, infections, or drug and antimicrobial treatments [6]. Thus, whether *Giardia* is a commensal or a parasite, it is conceivable that the perturbation of the host–parasite equilibrium may be the basis of some pathogenicity and may explain variations in symptoms both between hosts and within the same host over time [6]. If host microbiota may be primarily or secondarily involved in *Giardia* infection outcomes, many factors other than *Giardia* may affect the gut microbiome, potentially masking the Giardia microbiome relationship. The primary aim of this study was to investigate the impact of *Giardia* on gut microbiota in a homogeneous population of naturally infected dogs living in the same breeding facility. Moreover, the hematological, biochemical, and fecal parameters related to intestinal function were investigated.

## 2. Materials and Methods

### 2.1. Sample Description

Thirty-one German shepherd dogs living in the same breeding and training facility of the Italian Finance Police were included in the study. The dogs were housed in individual boxes and fed with the same commercial maintenance dry food (Crude protein: 29%, Crude fiber: 2%, Crude fat: 18%, Crude ash: 7.5%, Calcium: 1.3%). All dogs were annually vaccinated against the canine distemper virus (CDV), canine parvovirus (CPV), canine adenovirus (CAV), and Leptospirosis, regularly treated by anthelminthic drugs, and protected against ectoparasites using a slow-release insecticidal and repellent collar. The inclusion criteria were: (i) clinically healthy, without evidence of gastrointestinal disorders; (ii) no pharmacological therapy in the 2 months before the study; (iii) tested negative with a standard sedimentation-floatation coprological test. From each dog, the following samples were collected: a minimum of 10 g of fecal samples for parasitological and biochemical analysis; 2 independent biological replicates of rectal fecal swabs (FecalSwabTM, Copan Diagnostics Inc, Brescia, Italy) for microbial community analysis; 1 K3-EDTA and 1 plain tube of peripheral blood for hematological and biochemical investigations, respectively. Aseptic techniques and disposable equipment were used for each sample.

### 2.2. Giardia and Cryptosporidium spp. Detection and Quantification

The detection of *Giardia* and *Cryptosporidium* spp. was performed in fecal samples using the commercially available immunofluorescence test according to manufacturer instructions (Merifluor^®^ *Cryptosporidium*/*Giardia*, Meridian Bioscience, Cincinnati, OH, USA). Quantification of *Giardia* cysts and *Cryptosporidium* spp. oocysts was made by counting protozoa elements under the microscope and expressed as a number of (oo-) cysts per 1 g of fecal sample to a maximum of 50,000. Specimens with higher parasite (oo-) cysts were recorded in the report as >50,000.

### 2.3. Microbial Community Analyses

#### 2.3.1. DNA Extraction

Total DNA for metataxonomic analysis was extracted using a column-based kit (QIAamp DNA Mini Kit, QIAGEN, Hilden, Germany) starting from 200 μL of fecal sample in swab’s buffer (Modified Cary Blair medium), following the manufacturer’s instruction. Thermal lysis was carried out at 56 °C for 2 h, and RNaseA (70 Kunitz units/mg protein) was added to each sample to ensure RNA-free preparation. Total DNA was resuspended in 200 μL of nuclease-free water and stored at −20 °C until library preparation for sequencing.

#### 2.3.2. 16S rRNA Sequencing

Extracted DNA was used as a template in amplicon PCR to target the hypervariable V3 and V4 regions of the bacterial 16S rRNA gene. The amplification check was performed by 2% TAE agarose gel electrophoresis to identify a DNA fragment accounting for 550 bp length. The 16S library was prepared according to the Illumina 16S Metagenomic sequencing Library Preparation protocol, using the primers Bact341F and Bact785R (Fwd: CCTACGGGNGGCWGCAG and Rev:GACTACHVGGGTATCTAATCC) previously described by Klindworth A et al. [11] using the Nextera XT DNA Library Prep kit (Illumina, Milano, Italy). PCR clean-up was performed with Agencourt AMPure XP beads (Beckman Coulter Genomics, Indianapolis, IN, USA). Libraries were checked for both concentration and quality using Qubit and 2200 TapeStation (Agilent, Milano, Italy), respectively. Samples were equimolarity pooled, and sequencing was performed with an Illumina MiSeq platform using a MiSeq 600V3 cartridge (600 cycles, 2 × 300 bp, paired-end reads). Read sequences were deposited in the Sequence Read Archive (SRA) of the NCBI under the BioProject PRJNA736250.

#### 2.3.3. Reads Preprocessing and OTU Table Construction

After sequencing, data underwent a quality control procedure using the FastQC tool [12]. Data were then cleaned by removing adapters and primers and performing the dereplication of sequences using an in-house bash script. In addition, data were filtered based on the quality and length of the reads so that only reads with a quality higher than a given threshold (QPhred ≥ 20) and longer than 100 bp were retained. All subsequent steps were performed using QIIME2 pipeline version 2020.2 [13]. Raw sequence data were screened, trimmed, and denoised with DADA2 [14] (parameters: p-trunc-len-f = 288; p-trunc-len-r = 264) and quality filtered based on q-score. Operational taxonomic units (OTUs) were defined as sequences with at least 97% similarity with the Greengenes database (last release May 2013, version 13.8) [15]. Samples were rarefied to 98197 sequences per sample. The rarefaction depth was based on the lowest read depth of samples.

### 2.4. Hematological and Biochemical Analysis

Blood EDTA samples were used for complete blood cell count (CBC) with the hematology analyzer XN-1000 V (Sysmex Europe GmbH, Norderstedt, Germany), equipped with veterinary software (Software of Automated hematology Analyzer for Animal XN-V series (Sysmex). All samples were analyzed within 8 h after blood collection. Blood plain tubes were centrifuged 3000× *g* for 10 min, and serum samples were collected for biochemical analysis: routine biochemical profile analysis was performed using an automated clinical chemistry analyzer (Cobas C501; Roche Diagnostics International AG, Rotkreuz, Switzerland); folate and cobalamin (B12) immunoassay analysis was performed with an automated Cobas e601 analyzer (Roche Diagnostics). Serum canine C reactive protein (cCRP) concentration (mg/L) was determined via a commercially available turbidimetric immunoassay kit (Turbovet canine CRP, Acuvet Biotech, Zaragoza, Spain) applied to a Cobas c501 analyzer, following the manufacturer’s instructions. Fecal calprotectin was determined via a species-specific ELISA kit (Canine Calprotectin CP, MyBioSource, San Diego, CA, USA), following the manufacturer’s instructions. Briefly, 10 mg of samples were homogenized in 100 μL of PBS and centrifuged for 20 min at 1500× *g*; the supernatant was carefully collected and stored at −80 °C until analysis. Fecal samples were then thawed and measured in a unique batch.

### 2.5. Statistical Analyses

The overall goal of the statistical analysis was to compare the gut microbiota and the investigated hematological and biochemical parameters with respect to *Giardia* infection (*Giardia* Positive dogs (GP) and *Giardia* Negative dogs (GN)). Supposing a possible effect of the dog gender, the above-mentioned analysis was performed also considering the data stratified by the gender of the dogs. Therefore, the subgroups *Giardia*-Positive females (GPF), *Giardia*-Negative females (GNF), *Giardia*-Positive males (GPM), and *Giardia*-Negative males (GNM) were also evaluated in the statistical analysis. Due to the limited number of dogs, an additional stratification for age was not carried out. Nevertheless, the information about dogs’ age was used to check whether the investigated groups had a different age distribution by the Wilcoxon test. R 4.0.5 software [16], QIIme2 [13], and MetaboAnalyst 5.0 web portal [17] were used to conduct the statistical analysis. *p*-value < 0.05 was considered significant.

#### 2.5.1. Analysis of 16S rRNA Gene

Alpha diversity analysis was performed on the preprocessed count table and was measured by means of the Chao1 index to assess the richness. The Shannon index was used to assess the evenness and observed OTUs metrics to describe the community structure. The non-parametric Wilcoxon test was used to compare the alpha diversity and the OTUs number distribution between the GP and GN dogs. In addition, the non-parametric Kruskal–Wallis test was conducted to compare the GPF, GNF, GPM, and GNM groups. If significant, the pairwise comparison Wilcoxon test was performed by adjusting the *p*-value (padj-value) for multiple comparisons using the false discovery rate (FDR) method [18]. Beta diversity was evaluated with the phylogeny-based Unifrac distance metric. The PERMANOVA test (permutation number 999) was used to compare the beta diversity parameters among groups [19]. FDR correction for multiple testing was applied in pairwise comparisons between groups. The volcano plot [20] was drawn to identify the OTUs differing significantly between the groups of study. The features resulting in significance (*p*-value < 0.05) were selected and analyzed by partial least square discriminant analysis (PLS-DA) [21]. The most discriminating OTUs were shown in descending order of their coefficient scores. The ones with a coefficient score greater than 80 were identified and discussed.

#### 2.5.2. Hematological and BIOCHEMICAL Analyses

The volcano plot was built to identify the hematological and biochemical parameters that differ significantly between the groups of study. The significant variables (*p* < 0.05) were selected and described by means of a box plot.

## 3. Results

### 3.1. Sample Description

Overall, 19 female and 12 male German Shepherd dogs were included. The median age was 19 months (range: 15–85 months). Dogs ranged from 28 to 35 kg of body weight. All dogs were non-diarrhoeic and clinically healthy.

### 3.2. Giardia Detection and Quantification

Overall, 13 dogs were positive for *Giardia* (GP), of which 8 were females (GPF), and 5 were males (GPM). All positive dogs ranged from 15 to 24 months (Table S1), showing a significantly lower age distribution in *Giardia*-positive dogs than negatives (*p*-181 value = 0.0003). Equivalent conclusions were inferred considering the age distribution among females (padj-value = 0.021) and males (padj-value = 0.046). The same percentage of *Giardia* infection was observed among males and females (42%). The number of *Giardia* cysts per 1 g of fecal sample was higher than 50,000 in six dogs, between 10,000 and 50,000 in four dogs, and less than 10,000 in three dogs (Table S1). *Cryptosporidium* spp. was not detected in all the examined samples.

### 3.3. Effect of Giardia Infection on Gut Microbial Community Ecology

**Figure 1:**
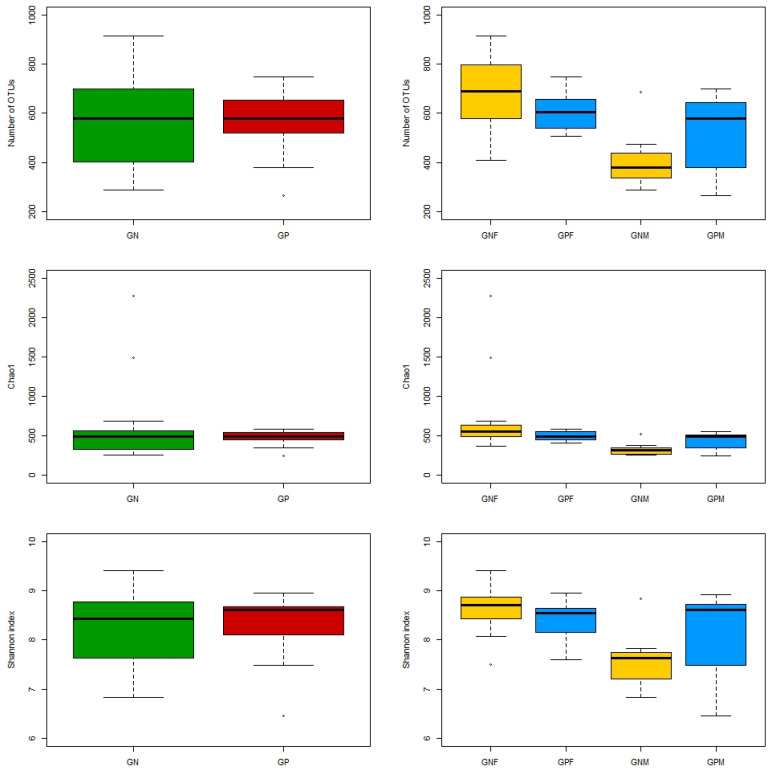
Box plot of the number of identified OTUs and Alpha-diversity (Chao1 and Shannon indices) by groups of study: GP and GN; GPF, GPM, GNF, and GNM. The box plot synthesize the data, providing the principal measures of central tendency and dispersion. Specifically, the diagram comprises a box with horizontal limits defining the upper and lower quantiles representing the interquartile range, with the median marked by a horizontal line within the box. The whiskers are vertical lines extending from the box as low as the 2.5th percentile and as high as the 97.5th percentile. Extreme values are indicated by dots.

After data preprocessing, a total of 705,154 reads (*n* = 31, mean for sample = 22,746.9, SD = 7254.902) were retained for bioinformatic analyses. The number of identified OTUs ranged between 200 and 600 if considering the entire sample with the exception of dog number 9, presenting a number of identified OTUs higher than 1000. No significant differences were found in the number of OTUs between GP and GN. Focusing on gender, no significant differences were found between GPF and GNF and between GPM and GNM. Considering the positive dogs, no significant differences were found in the number of OTUs between GPF and GPM, while the number of OTUs was significantly higher in GNF than in GNM (padj-value = 0.011) (Figure 1). Alpha diversity was analyzed using Chao1 and Shannon indices. Chao1 indices were 470.11 ± 94.93 (GP), 596.93 ± 501.66 (GN), 495.80 ± 60.66 (GPF), 429.88 ± 129.96 (GPM), 765.38 ± 584.98 (GNF) and 332.22 ± 94.70 (GNM) while Shannon indices were 7.90 ± 0.73 (GP), 7.89 ± 0.78 (GN), 8.07 ± 0.45 (GPF), 7.65 ± 1.06 (GPM), 8.32 ± 0.5 (GNF), and 7.20 ± 0.64 (GNM). No statistically significant difference between GP and GN groups with respect to both Chao1 and Shannon indices was observed. The same result was found when comparing GPF and GNF and between GPM and GNM. Focusing on positive dogs, no statistically significant difference was observed between GPF and GPM for both tested indices, whereas comparing GNF and GNM, the alpha diversity indices 204 Chao1 and the Shannon were significantly higher in GNF than GNM (padj-value = 0.0057 and padj-value = 0.043, respectively) (Figure 1). The unique OTUs of different groups were summarized according to the result of the OTU clustering analysis in Table 1.

**Figure 2:**
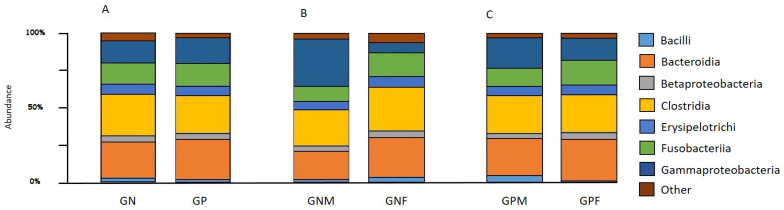
Barplots showing the average abundance of bacterial taxa at the order level between (**A**) GN and GP dogs, (**B**) GNM and GNF, and (**C**) GPM and GPF.

The OTUs table is reported in Table S2. The beta diversity index between the GP and GN groups was significantly different (*p*-value = 0.0025). The same results were found in the comparison between GNF and GPF (padj-value = 0.02), whereas no statistically significant difference was observed between GNM and GPM. Focusing on negative dogs, the beta diversity index was different between the GNF and GNM groups (padj-value = 0.02), while no statistically significant differences were found between GPF and GPM groups. To display the proportion of different taxa at the class level, the bar plot reported in Figure 2 was generated based on the relative abundance of taxa. The results showed that 90% of microorganisms in the fecal samples of the entire dataset belonged to seven classes (Bacilli, Bacteroida, Betaproteobacteria, Clostridia, Eryspelotrichi, Fusobacteria, Gammaproteobacteria) (Figure 2A). The comparison between GNF and GNM groups revealed the presence of three classes unevenly distributed: Gammaproteobacteria, Bacteroida, and Fusobacteria. The Gammaproteobacteria were found with a higher frequency in the GNM group than the GNF one; on the contrary, the two classes of Bacteroida and Fusobacteria displayed a higher frequency in GNF than in GNM (Figure 2B). A very similar distribution in the relative taxa frequency was found between the GPF and GPM groups (Figure 2C).

**Figure 3:**
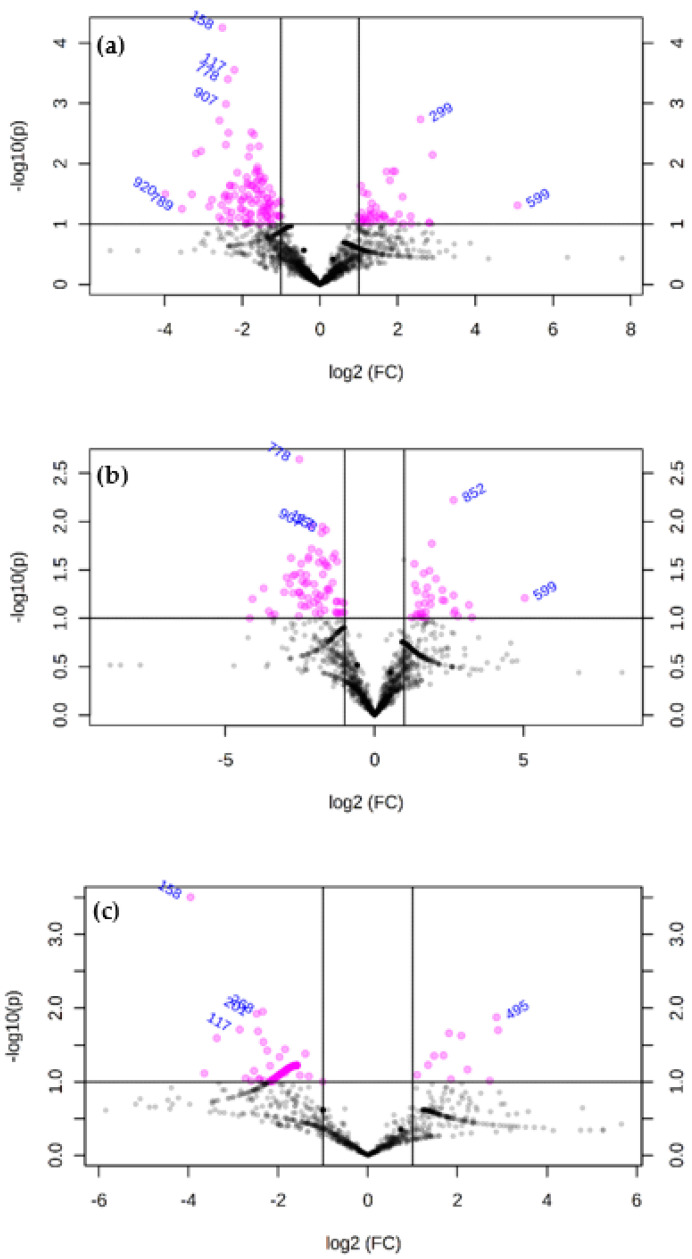
Volcano plot showing the OTUs that differ significantly between groups of study: (**a**) GP and GN, (**b**) GPF and GNF, and (**c**) GPM and GNM. The pink points indicate variables of interest that display both large-magnitude fold changes (*x*-axis) as well as high statistical significance (−log10 of *p*-value Wilcoxon test, *y*-axis). The horizontal line shows the *p*-value cut-off (*p*-value = 0.10), with points above the line having a *p*-value < 0.10 and points below the line having a *p*-value > 0.1. The vertical lines show 2-fold changes.

Figure 3 reports the results of volcano plots identifying the OTUs differing significantly (*p* < 0.05) between GN and GP (a), GNF and GPF (b), and GNM and GPM (c).

**Figure 4:**
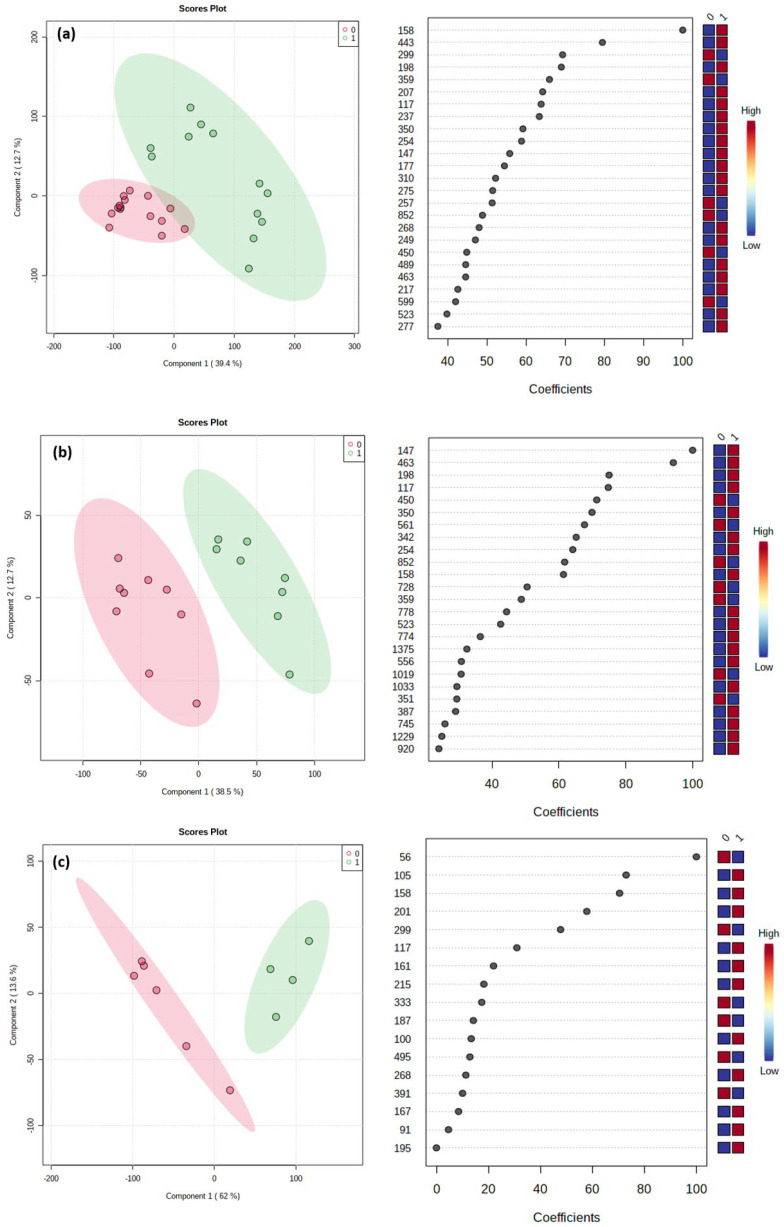
Partial least squares discriminant analysis (PLS-DA) results showing the comparison between OTU data acquired for (**a**) GP vs. GN, (**b**) GPF vs. GNF, and (**c**) GPM vs. GNM. On the left, the 2-D PLS-DA scores plots; on the right, the variable importance in projection plots. The most discriminating OTUs are shown in descending order of their coefficient scores. The color boxes indicate whether OTU is increased (red) or decreased (blue) in positive (1) vs. negative (0).

The outputs of the PLS-DA analysis, applied to the previously identified significant OTUs, were shown using the scores plots and the variable importance in projection plots (Figure 4).

Moreover, in the supplementary results, the volcano plots and the outputs of the PLS-DA applied to GPF and GPM (a) and GNF and GNM (b) are shown (Figures S1 and S2, respectively). All 228 plots displayed a good discriminatory power, explaining from more than 50% (Figure 4a) to about 80% (Figure S2b) of the total variance of the models. The most relevant variables, corresponding to coefficient values greater than 80 for each of the five pairwise comparisons, were reported in Table 2.

The analysis revealed significant differences in the relative abundance of specific taxa between positive and negative dogs with specific reference to *Bacteroidales* order that was found more abundant in *Giardia*-positive dogs. In addition, when the positive and negative groups were broken down by gender, significant differences emerged. Specifically, within the female group, GP dogs displayed more abundance of *Erysipelotrichales* and *Bacteroidales* orders than GN ones. Within the male group, GN dogs were mainly colonized by *Clostridiales* than GP. Comparing gender, GPM showed a higher abundance of *Clostridiales* and *Lactobacillales* with respect to GPF and a higher abundance of *Erysipelotrichales* was found in both GPM and GPF, probably referring to different families or genera. Regarding GNF, they were characterized by a higher abundance of *Burkholderiales* compared to the GNM group, which displayed a higher abundance of *Clostridiales* compared to GNF (Table 2).

### 3.4. Hematological and Biochemical Analysis

**Figure 5:**
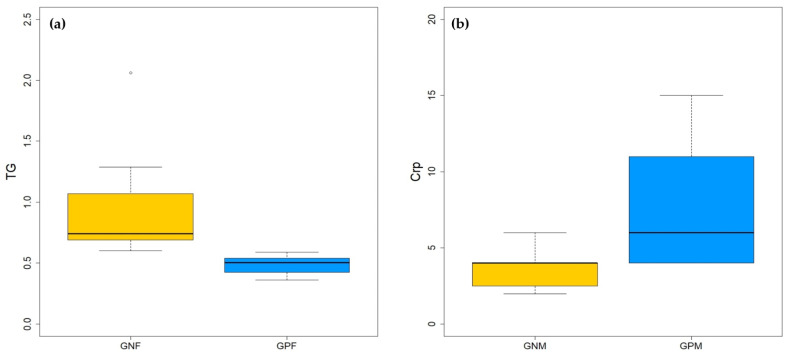
Box plot of the significant hematological and biochemical parameters in (**a**) female and (**b**) male dogs. TG is expressed in mmol/L, CRP in mg/L.

All the tested hematological and biochemical parameters were within the range of normal values in all dogs (Table S1). The volcano plot analysis showed no significant differences in hematological and biochemical parameters between GP and GN dogs. Focusing on gender subgroups, statistically significant higher levels of Triglycerides (TG) were observed among GNF if compared with GPF (Figure 5a, *p*-value = 0.0051), while, at the significance limit, GPM had higher levels of cCRP than GNM (Figure 5b, *p*-value = 0.0725). Considering the *Giardia*-negative dogs, no differences were observed in the hematological and biochemical parameters between males and females. Instead, among the *Giardia*-positive dogs, GPF had significantly higher levels of lipase than GPM (p251 value = 0.0429) and significantly lower levels of cCRP (*p*-value = 0.0204) (Figures S3 and S4).

## 4. Discussion

The primary aim of this study was to investigate possible variations in gut microbiota in a population of asymptomatic dogs, naturally infected or not by *Giardia*; secondarily, we investigated possible variations in some hematological, biochemical, and fecal parameters with respect to *Giardia* infection. Few previous studies aimed to investigate the relationship between *Giardia* and gut microbiota structure and composition in dogs [22,23,24,25]. However, the majority of these studies were affected by different possible confounders such as uncontrolled lifestyles (stray dogs), different origins or breeding of dogs, concurrent parasitic infections, different clinical signs, and anthelmintic treatments [22,23,25]. In order to minimize potential biases, this study was performed on a homogeneous population belonging to the same breed, housed in the same conditions, and fed with the same commercial maintenance dry food. Moreover, all dogs included in the present study were clinically healthy, no therapies against *Giardia* or bacteria were administered in the previous two months, and coprological investigations excluded co-infection with other intestinal parasites. In our survey, the overall percentage of *Giardia* infection was 42%, with no differences between male and female dogs. Prevalence rates of *Giardia* infection in dogs vary depending on the population under study and diagnostic method used and can be as high as 45% [26]. Similar prevalence in the shelter and commercial kennels were previously reported [4,5,27,28]. We found that infected dogs were younger than negative ones, and this was an expected finding since previous reports showed that younger dogs have a higher risk of *Giardia* infection than older ones [6,28,29,30,31,32]. Similarly, it has been demonstrated that children are more likely to suffer from clinical infections than adults [33]. Differences in susceptibility to *Giardia* infection among different age groups are likely due to age-related shifts in microbiota composition as well as the variability of the hosts’ immune factors [34]. Even though the precise mechanisms that undergo *Giardia* pathogenesis are incompletely understood, a pivotal role has been postulated for microbiota by recent research [35]. Functional and compositional modifications of gut microbiota have been demonstrated during the course of *Giardia* infection with particular reference to changes in microbial community biodiversity and altered species abundance [36]. In our investigation, some significant differences in terms of microbial diversity were observed among tested groups. Particularly *Giardia* infection was associated with a significant shift of beta diversity substantiated by a relevant reduction of Gammaproteobacteria and an increase of Fusobacteria in GPM if compared with GNM. This also highlights a reduction of the differences in terms of microbial abundance between males and females in the presence of the infection. The reduction of Gammaproteobacteria and the increase of Fusobacteria have been observed in other experimental settings with regard to positive asymptomatic dogs [24]. Moreover, PLS-DA analysis displayed a significant imbalance of different bacterial taxa with particular reference to OTUs referred to as the *Erysipelotrichales*, *Lactobacillales*, *Clostridiales*, and *Burkholderiales* orders, with the first two being present with a greater extent in *Giardia*-positive dogs. Both *Erysipelotrichales* and *Lactobacillales* orders are of particular interest in this specific context. The occurrence of *Erysipelotrichales* in *Giardia*-positive dogs could be strongly related to the progression of the infection. Indeed, many researchers have addressed the importance of *Erysipelotrichaceae* in inflammation-related disorders of the gastrointestinal tract so far, with particular reference to the human context. *Erysipelotrichaceae* abundance levels were found to be increased in the lumen of colorectal cancer patients as compared to healthy controls [37] and to be significantly higher in the tumor group of an animal model of 1, 2-dimethylhydrazine-induced colon cancer [38]. However, high inter-host variation has been observed so far [35], probably due to the inherent differences in species related to the gut microbiota (i.e., mice and humans) and/or differences in the immune responses upon sensing bacterial ligands [39,40]. Moreover, many studies have demonstrated the association between this bacterial order and host lipid metabolisms [37,38,41,42,43,44]. Regarding the order of *Lactobacillales*, their involvement in contrasting infection-induced gut oxidative stresses [45] and preventing the adherence of *Giardia* trophozoites to the mucosal surface are well known [46]. These findings draw a possible scenario in which the microbiota of *Giardia*-positive subjects, on the one hand, metabolically supports the persistence of *Giardia* by means of *Erysipelotrichaceae* abundance and on the other hand, counteracts the host’s inflammation caused by *Giardia* itself by means of *Lactobacillales* action. In our study, all the biochemical parameters were within the normal ranges, and significant differences were observed between *Giardia*-positive and negative dogs with respect to gender. Particularly, among *Giardia*-positive dogs, serum lipase was significantly higher in females than in males. Moreover, GPF showed significantly lower values of triglycerides than *Giardia*-negative females. These findings may be linked to *Giardia*’s limited ability to synthesize lipids for membranes and organelles biosynthesis, energy production, and growth [47]. Thus, lipids have to be provided by the host itself and/or its gut microbiota through the conversion of primary bile acids to secondary bile acids [48]. As a consequence, the host lipid metabolism during giardiosis might have a key role in keeping the parasite persistent in the intestinal lumen [24]. When a host ingests the cysts, these enter the digestive tract, where they are stimulated by the acidic milieu in the stomach and the presence of bile and trypsin in the duodenum to develop into motile trophozoites in the proximal small intestine. In the upper intestinal tract, the trophozoites proliferate and use their adhesive disc to attach to the intestinal villi [49]. As the parasite density increases and the trophozoites descend into the lower intestinal tract, they encounter decreased cholesterol, an increase in pH, and increased concentrations of bile and lactic acid [50]. These conditions promote trophozoite differentiation into infectious cysts that are released into the environment [50]. Moderately-enhanced bile acid concentration has been found to promote *Giardia* growth in vitro [48]. The mechanism by which bile stimulates parasite growth is unknown, but uptake of conjugated bile salt by *Giardia* could reduce intraluminal bile salt concentrations and possibly interfere with micellar solubilization of fat and *Giardia*–bile salt interactions in vitro and in vivo [51]. Moreover, *Giardia* infection is associated with malabsorption of fats due to mechanical mucosal damage, leading to intestinal steatosis and increased transit of lipids into the distal small intestine and colon [51]. In our study, we investigated different functional (cobalamin, folate) and biochemical (C-reactive protein, calprotectin, alkaline phosphatase) biomarkers that have been shown to be useful indicators of intestinal inflammation in dogs [52]. Interestingly, *Giardia*-positive males displayed significantly higher values of cCRP than negative males as well as positive females. Even though values remained within the reference range, this finding supports the presence of a pro-inflammatory state in these subjects. Differently, no significant differences among groups were observed in the values of the other investigated parameters. Taken together, these findings are coherent with the absence of clinical symptoms and allow us to exclude severe gastro-intestinal inflammation in the investigated dogs, reinforcing the possibility of a delicate *Giardia*–microbiome–host equilibrium that may prevent clinical manifestations.

## 5. Conclusions

Investigating *Giardia* ecological milieu in relation to the host’s resident microbial community and its metabolic context is a very challenging task as it requires taking into consideration homogeneous cohorts that do not show evident signs of disease, such as diarrheal syndrome and intestinal malabsorption, known to compromise the luminal microbial environment and the serum biochemical profile. Here, we report for the first time a study comparing a group of *Giardia*-negative dogs with a group of *Giardia*-positive but asymptomatic subjects. The main strength of this study is the lack of confounders, such as different breeding and nutrition of dogs, concurrent parasitic infections, and clinical signs. On the other hand, a possible limit is represented by the low number of dogs considered, which may have prevented us from observing further differences in the study groups. Our results showed that the presence of *Giardia* exerts an effect upon the gut microbial communities, enriched in protective taxa against gut inflammation and depleted in lipid-producing taxa, potentially usable to limit *Giardia* infection and relieve host inflammation. Taking together the outcomes of our study suggests that treatments against *Giardia* should be considered with caution in asymptomatic subjects in order to save the *Giardia*–microbiome–host equilibrium.

## Figures and Tables

**Table 1:** Unique OTUs per group.

Groups	GP	GN	GPF	GPM	GNF	GNM
N.OTUs	2112	4863	754	652	3462	640

**Table 2:** Differential abundance of microbial taxa among groups. In the “Group” column are reported the groups with higher abundance. GP = *Giardia*-positive; GN = *Giardia*-Negative; GPF = *Giardia*-Positive Females; GPM = *Giardia*-Postive Males; GNF = *Giardia*-Negative Females; GNM = *Giardia*-Negative Males.

ID	Phylum	Class	Order	Group
GN vs. GP				
158	*Bacteroidetes*	*Bacteroidia*	*Bacteroidales*	GP
443	*Bacteroidetes*	*Bacteroidia*	*Bacteroidales*	GP
GNF vs. GPF				
147	*Firmicutes*	*Erysipelotrichi*	*Erysipelotrichales*	GPF
463	*Bacteroidetes*	*Bacteroidia*	*Bacteroidales*	GPF
GNM vs. GPM				
56	*Firmicutes*	*Clostridia*	*Clostridiales*	GNM
GNF vs. GNM				
45	*Proteobacteria*	*Betaproteobacteria*	*Burkholderiales*	GNF
52	*Proteobacteria*	*Betaproteobacteria*	*Burkholderiales*	GNF
56	*Firmicutes*	*Clostridia*	*Clostridiales*	GNM
GPF vs. GPM				
885	*Firmicutes*	*Clostridia*	*Clostridiales*	GPM
124	*Firmicutes*	*Erysipelotrichi*	*Erysipelotrichales*	GPM
201	*Firmicutes*	*Clostridia*	*Clostridiales*	GPM
238	*Firmicutes*	*Clostridia*	*Clostridiales*	GPM
105	*Firmicutes*	*Bacilli*	*Lactobacillales*	GPM
613	*Firmicutes*	*Erysipelotrichi*	*Erysipelotrichales*	GPF

## Data Availability

The datasets supporting the conclusions of this article are available in the NCBI repository, BioProject n. PRJNA736250 (https://www.ncbi.nlm.nih.gov/bioproject/PRJNA736250).

## Supplementary Materials

The following supporting information can be downloaded at: https://www.mdpi.com/article/10.3390/ani13060958/s1, Table S1: complete analysed dataset with metadata; Figure S1: Volcano plot showing the OTUs that differ significantly between groups of study; Figure S2: Partial least squares discriminant analysis (PLS-DA) results showing the comparison between OTU data acquired; Figure S3: Volcano plot showing the hematological and biochemical parameters that differ significantly between groups GPF and GPM; Figure S4: Box plot of the significant hematological and biochemical parameters in positive dogs by gender.
