# Supplementary material for: Giardia duodenalis Colonization Slightly Affects Gut Microbiota and Hematological Parameters in Clinically Healthy Dogs
Source: Animals (Basel). 2023 Mar 7;13(6):958. doi: 10.3390/ani13060958 (PMC10044607; doi:10.3390/ani13060958)
Supplement: Supplementary file 1 [file animals-13-00958-s001.zip › animals-2207089-supplementary/animals-2207089-supp Figures.docx]

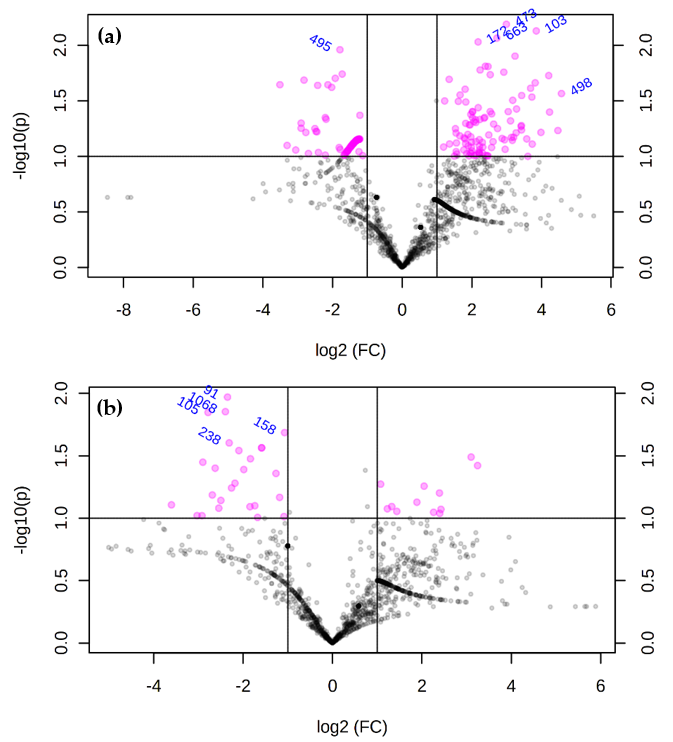


**Figure S1.** Volcano plot showing the OTUs that differ significantly between groups of study: (**a**) GNF and GNM (**a**) and (**b**) GPF and GPM. The pink points indicate variables of interest that display both large-magnitude fold changes (*x*-axis) as well as high statistical significance (−log10 of *p*-value Wilcoxon test, *y*-axis). The horizontal line shows the *p*-value cut-off (*p*-value = 0.10), with points above the line having a *p*-value < 0.10 and points below the line having a *p*-value > 0.1. The vertical lines show 2-fold changes.


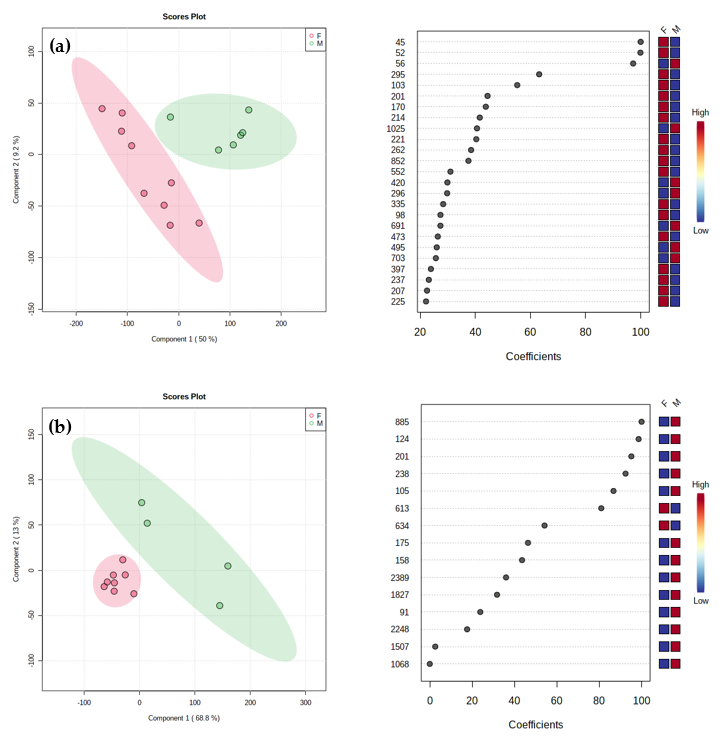


**Figure S2.** Partial least squares discriminant analysis (PLS-DA) results showing the comparison between OTU data acquired for (**a**) GNM vs. GNF and (**b**) GPM vs. GPF. On the left, the 2-D PLS-DA scores plots; on the right, the variable importance in projection plots. The most discriminating OTUs are shown in descending order of their coefficient scores. The color boxes indicate whether OTU is increased (red) or decreased (blue) in females (F) vs. males (M).


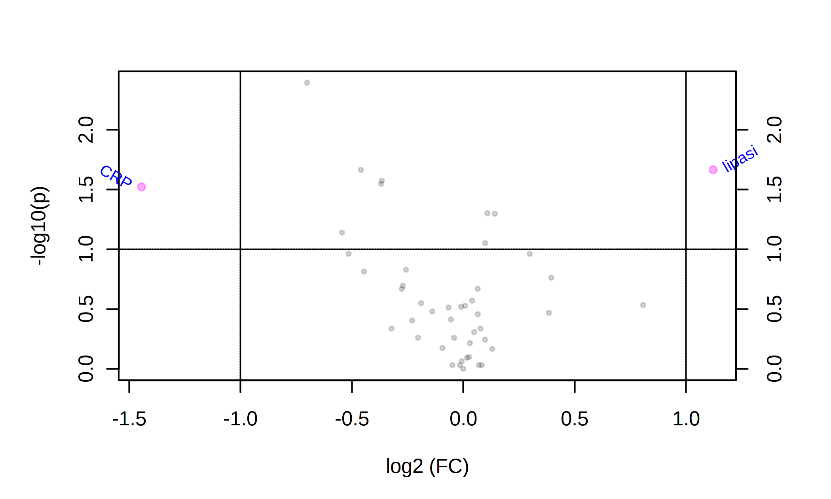


**Figure S3.** Volcano plot showing the hematological and biochemical parameters that differ significantly between groups GPF and GPM. The pink points indicate variables of interest that display both large-magnitude fold changes (*x*-axis) as well as high statistical significance (−log10 of *p*-value Wilcoxon test, *y*-axis). The horizontal line shows the *p*-value cut-off (*p*-value = 0.10), with points above the line having a *p*-value < 0.10 and points below the line having a *p*-value > 0.1. The vertical lines show 2-fold changes.


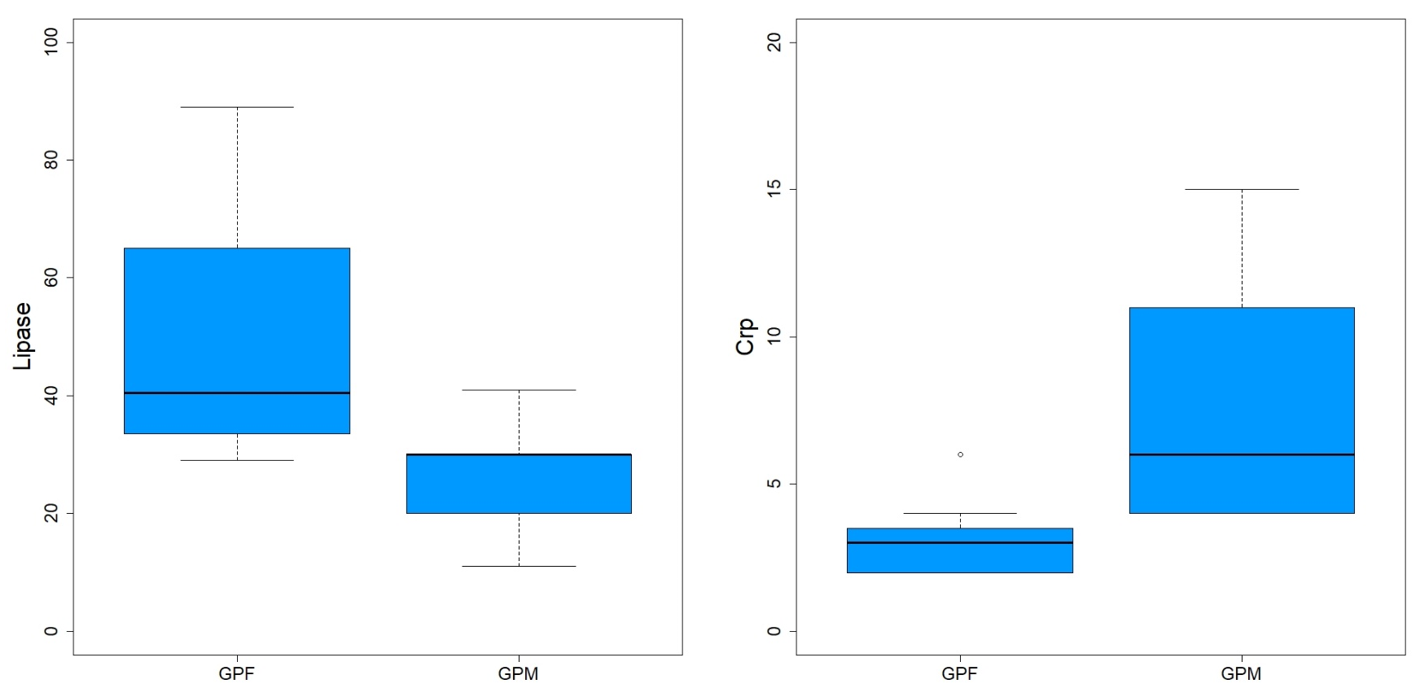


**Figure S4.** Box plot of the significant haematological and biochemical parameters in positive dogs by gender. The box plot synthesizes the data, providing the principal measures of central tendency and dispersion. Specifically, the diagram comprises a box with horizontal limits defining the upper and lower quantiles representing the interquartile range, with the median marked by a horizontal line within the box. The whiskers are vertical lines extending from the box as low as the 2.5th percentile and as high as the 97.5th percentile. Extreme values are indicated by dots.
